# Noisy Galvanic Vestibular Stimulation Modulates the Amplitude of EEG Synchrony Patterns

**DOI:** 10.1371/journal.pone.0069055

**Published:** 2013-07-18

**Authors:** Diana J. Kim, Vignan Yogendrakumar, Joyce Chiang, Edna Ty, Z. Jane Wang, Martin J. McKeown

**Affiliations:** 1 Neuroscience, University of British Columbia, Vancouver, Canada; 2 Pacific Parkinson’s Research Centre, Vancouver, Canada; 3 Department of Electrical and Computer Engineering, University of British Columbia, Vancouver, Canada; 4 Department of Medicine (Neurology), University of British Columbia, Vancouver, Canada; 5 Brain Research Centre, University of British Columbia, Vancouver, Canada; Hangzhou Normal University, China

## Abstract

Noisy galvanic vestibular stimulation has been associated with numerous cognitive and behavioural effects, such as enhancement of visual memory in healthy individuals, improvement of visual deficits in stroke patients, as well as possibly improvement of motor function in Parkinson’s disease; yet, the mechanism of action is unclear. Since Parkinson’s and other neuropsychiatric diseases are characterized by maladaptive dynamics of brain rhythms, we investigated whether noisy galvanic vestibular stimulation was associated with measurable changes in EEG oscillatory rhythms within theta (4–7.5 Hz), low alpha (8–10 Hz), high alpha (10.5–12 Hz), beta (13–30 Hz) and gamma (31–50 Hz) bands. We recorded the EEG while simultaneously delivering noisy bilateral, bipolar stimulation at varying intensities of imperceptible currents – at 10, 26, 42, 58, 74 and 90% of sensory threshold – to ten neurologically healthy subjects. Using standard spectral analysis, we investigated the transient aftereffects of noisy stimulation on rhythms. Subsequently, using robust artifact rejection techniques and the Least Absolute Shrinkage Selection Operator regression and cross-validation, we assessed the combinations of channels and power spectral features within each EEG frequency band that were linearly related with stimulus intensity. We show that noisy galvanic vestibular stimulation predominantly leads to a mild suppression of gamma power in lateral regions immediately after stimulation, followed by delayed increase in beta and gamma power in frontal regions approximately 20–25 s after stimulation ceased. Ongoing changes in the power of each oscillatory band throughout frontal, central/parietal, occipital and bilateral electrodes predicted the intensity of galvanic vestibular stimulation in a stimulus-dependent manner, demonstrating linear effects of stimulation on brain rhythms. We propose that modulation of neural oscillations is a potential mechanism for the previously-described cognitive and motor effects of vestibular stimulation, and noisy galvanic vestibular stimulation may provide an additional non-invasive means for neuromodulation of functional brain networks.

## Introduction

The vestibular system may be considered a sixth sense [1] but thalamic and cortical processing of vestibular sensory information is especially complex, multimodal and widespread. While the parieto-insular vestibular cortex has been described as the “core” vestibular region in non-human primates [2], present views gravitate towards the notion of a highly distributed vestibular network comprising the lateral and medial frontal cortices, somatosensory cortex, premotor region, temporo-parietal junction, posterior parietal cortex, anterior and posterior insula, hippocampus and cingulate cortex [3], [4]. The widespread nature of vestibular projections is mediated by multiple vestibular-responsive thalamic nuclei and corticothalamocortical communication [3], [5]–[8]. Our understanding, however, of the vestibular influences on cortical and subcortical networks remains incomplete.

Galvanic vestibular stimulation (GVS) confers many advantages for investigating the effect of vestibular input on brain function. Transcutaneous delivery of galvanic current to the mastoid processes alters firing rates of vestibular afferents though, unlike natural or caloric stimuli, without canal or otolithic directional specificity [9], [10]. Nevertheless, direct and precisely controlled perturbation of the vestibular system using GVS has facilitated the modern study of balance, dynamic movements and cognitive effects while largely avoiding unwanted side effects of vertigo, nausea and nystagmus [11]–[15]. Therefore, due to the usefulness and tolerability of GVS, growing interest has expanded its role in neuropsychological and neurorehabilitation purposes for both normal and patient groups [16]. For example, with application of noisy (i.e., with random fluctuations) GVS, studies have demonstrated enhancement of cognitive abilities, such as visual memory, in healthy subjects [12]. Noisy GVS applications have also extended to neurological diseases, with evidence suggesting stimulation improves hemispatial neglect and prosopagnosia in stroke patients [15], [17] while caloric vestibular stimulation has been shown to alleviate neuropathic pain [18], [19]. Additionally, Yamamoto et al. delivered noisy GVS in the context of motor tasks to patients with central neurodegenerative disorders, including Parkinson’s disease [20]. Patients improved in their motor responsiveness during periods of stimulation, an outcome that has been subsequently reproduced [21], [22], although, like the previously stated cognitive findings, the mechanism remains largely unexplained.

The reported motoric benefit of noisy GVS in Parkinson’s disease patients is particularly intriguing considering that the Parkinsonian state is characterized by highly synchronized beta oscillations (15–30 Hz), which propagate throughout a basal ganglia-thalamocortical network. These predominantly low-frequency oscillations (>30 Hz) have been recorded in the external globus pallidus (GPe), subthalamic nucleus (STN), striatal and M1 neurons in dopamine-depleted animal models [23]–[25]. Oscillatory synchronization below 30 Hz has similarly been observed in local field potential (LFP) recordings of STN, internal (GPi) and external pallidal (GPe) neurons in patients off medication [26]–[28]. In corroboration with these findings, sensorimotor EEG potentials recorded from Parkinson’s disease patients have been observed to strongly resonate at 20 Hz, and to a lesser extent at 10 Hz [29]. Partially driven by a pattern generator comprised of the STN-GPe network [30], the exaggerated entrainment of neurons in the beta band throughout a basal ganglia-thalamocortical network has been suggested to serve as a basis for bradykinesia and movement impairments [30], [31]. However, the exact manner in which beta synchrony affects sensorimotor processing is currently contentious. Previous studies have suggested that the high beta synchrony observed in Parkinson’s disease may be “antikinetic” or may prevent processing of novel information, thereby accounting for poverty of movement [32]–[34]. Yet, recent evidence suggests that the beta synchronization observed in the dopamine-depleted and Parkinsonian condition is indicative rather of a functional network “stuck” in one of many normal dynamic states [35].

Given the previously stated cognitive and behavioural effects of GVS, we hypothesized noisy GVS will alter neural oscillatory dynamics, particularly in the beta band. The ability of GVS to modulate slower delta and theta brain rhythms during a visual processing task has been demonstrated before in healthy subjects, although using a direct current stimulus [36]. Since variable levels of noise may optimize incoming signal detection and neural transmission [37] and in consideration of the previously stated cognitive and behavioural effects using noisy GVS [12], [15], [17], [20]–[22], we were particularly interested in whether noisy stimulation modulates EEG rhythms. External influences via non-invasive brain stimulation techniques on the oscillatory dynamics of the brain is highly relevant since the brain uses neural synchronization as a mechanism to dynamically shift between transient functional network states [38], [39]. Generally speaking, oscillatory dynamics and synchrony patterns, which have been demonstrated to temporally coincide with perceptual cues [40]–[42], are therefore hypothetically associated with information transmission relevant for a particular behaviour or function [38]. For example, using normal, intact rats, Leventhal et al. demonstrated that beta synchronization reflects a post-decision state of motor output decision following a sensory cue [35]. Furthermore, in addition to Parkinson’s disease, abnormal oscillations characterize numerous neurological and neuropsychiatric disorders such as neurogenic pain, tinnitus and depression [43]. Therefore, the effect of GVS and noisy sensory input on ongoing – normal and pathological – brain oscillatory dynamics is an unaddressed issue of further interest.

Here we investigated whether imperceptible, noisy GVS is capable of modulating standard EEG rhythms in normal, healthy subjects. We applied a noisy stimulus with 1/*f* power features, and investigated the subsequent effect on recordings within theta (4–7.5 Hz), low alpha (8–10 Hz), high alpha (10.5–12 Hz), beta (13–30 Hz) and gamma (31–50 Hz) bands. We investigated the transient aftereffects and simultaneous neural changes during eyes-open resting state as a result of transcranial noisy vestibular stimulation. Previously, large stimulus-based EEG artifacts have disrupted the ongoing measurement of microvolt-level brain oscillations, a complication presently circumvented by: 1) improved EEG amplifier design with high common-mode-rejection ratio, and 2) a combination of well-established artifact rejection and factorization analytical techniques such as Independent Component Analysis (ICA) [44] and the QR decomposition [45], [46]. Using subsequent power spectral analysis and Least Absolute Shrinkage Selection Operator (LASSO) regression, we aim to demonstrate whether noisy GVS is associated with changes in the amplitude of oscillatory synchrony patterns that are due to both direct and ongoing effects. We show an immediate and brief suppression of gamma power in lateral regions after stimulation stopped; additionally, after cessation of GVS, we observed a delayed increase (after ∼20–25 s) in beta and gamma power in frontal regions, altogether indicating a global and direct effect of noisy vestibular stimulation on EEG rhythms. More importantly, using LASSO regression, we show that noisy GVS modulates the power of ongoing EEG synchrony across theta, alpha, beta and gamma bands, providing evidence of its ability to directly influence brain rhythms.

## Materials and Methods

### Subjects

Ten healthy individuals (five males, five females; aged from 20 to 63 years; mean age 37.2±17.7 years; all right-handed) without any reported vestibular, auditory or neurological disorders participated in the study. Since the present study was novel and exploratory, we selected a range of young and older adults in order to preclude potential age-dependent factors that may bias our results. Data for one participant were excluded due to excessively noisy, corrupted data (<50% data yield).

### Ethics Statement

The study was approved by the University of British Columbia Clinical Research Ethics Board. All subjects gave written, informed consent prior to participation. Research was conducted according to the principles expressed in the Declaration of Helsinki.

### Primary Study Protocol

Subjects were comfortably seated 80 cm from a screen, and were instructed to focus their gaze on a continuously displayed fixed target to minimize distractions while the EEG was recorded (6 trials, 192 s each). In each trial, EEG was first recorded without stimulation for 60 s (pre-stimulus period), blinding subjects to the actual stimulus onset. Noisy stimulation signals were then delivered for a fixed duration of 72 s (stimulation period), followed by a sham current for 60 s (post-stimulus period). During the stimulation period within each trial, we applied one of six imperceptible currents: 10, 26, 42, 58, 74 and 90% of the determined threshold value. For each subject, the delivery of the 6 trials and respective stimulation intensities were differently permutated in a pseudorandom order.

### Stimulus

**Figure 1:**
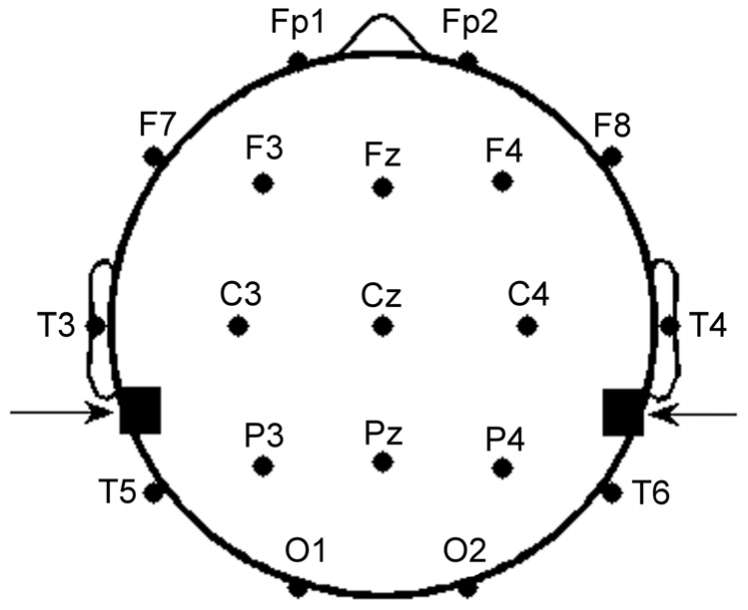
Placement of EEG and stimulating electrodes. 19 recording electrodes were placed on the scalp according to the International 10–20 System. Galvanic vestibular stimulation (GVS) electrodes were placed with one electrode on the mastoid process behind each ear (denoted by arrows) for bilateral configuration and transmastoidal stimulation.

GVS was delivered to subjects through carbon rubber electrodes (17 cm^2^) in bilateral, bipolar fashion. For bilateral stimulation, an electrode was placed over the mastoid process behind each ear (Figure 1), and coated with Tac gel (Pharmaceutical Innovations, NJ, USA) to optimize conductivity and adhesiveness. Digital signals were generated on a computer with Labview software and converted to analog signals via a NI USB-6221 BNC digital acquisition module (National Instruments, TX, USA). The analog command voltage signals were subsequently passed to a constant current stimulator (Model DS5, Digitimer, Hertfordshire, UK), which was connected to the stimulating electrodes.

**Figure 2:**
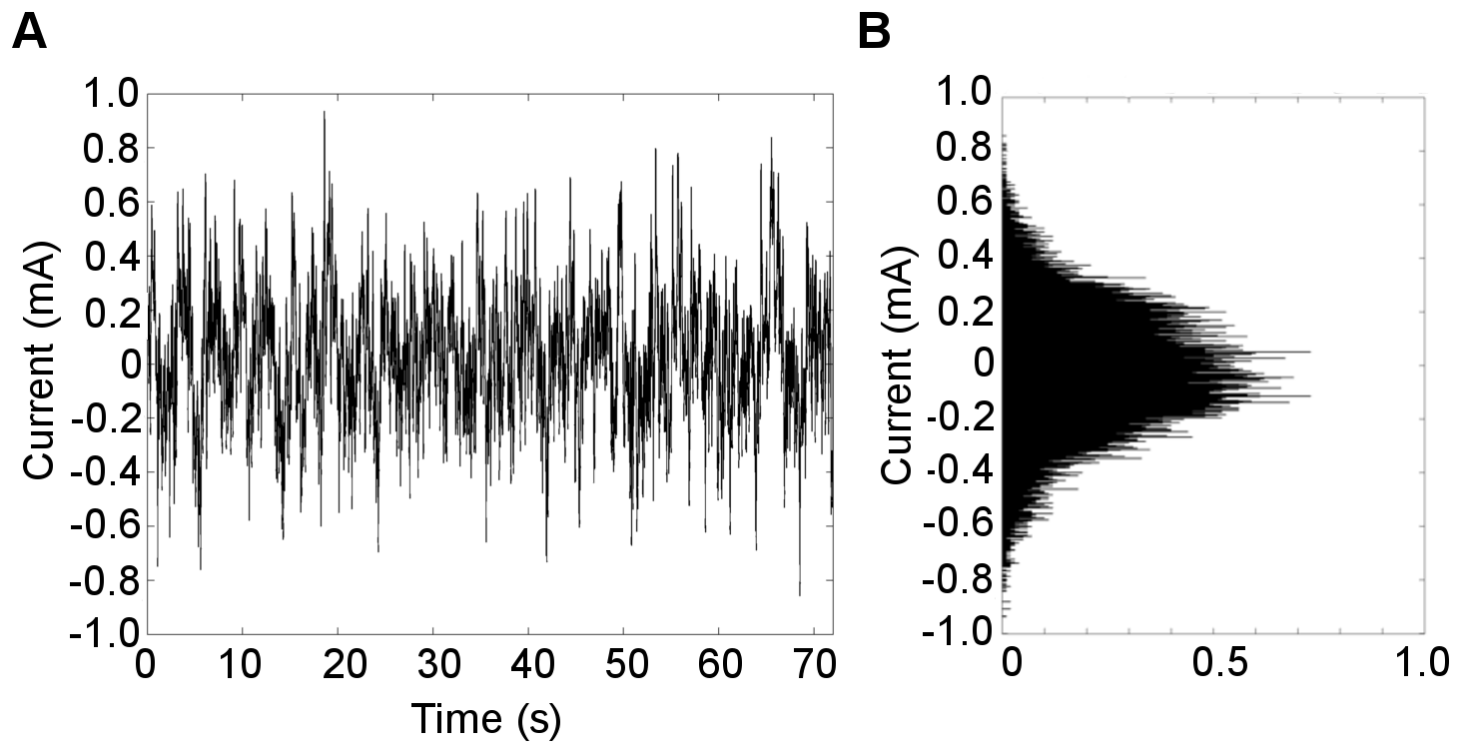
Characteristics of the stimulus. **A.** Typical recording from a subject receiving a noisy stimulus applied for 72 s duration. The stimulus presented is at the highest current intensity (current level 6), which was set to 90% of the subject’s individual sensory threshold (RMS current value of 242 µA). **B.** Probability density function of the stimulus current follows a Gaussian distribution.

Bipolar stimulation signals were zero-mean, linearly detrended, noisy currents with a 1/*f*-type power spectrum (pink noise) as has been previously applied in Parkinson’s disease and healthy subjects [20], [21], [47]. The stimulation signal was generated between 0.1–10 Hz with a Gaussian current density, with the command signal delivered to the constant-current amplifier at 1 kHz (Figure 2). The stimulus was applied at an imperceptible level to avoid effects by general arousal and/or voluntary selective attention, with the current level individually determined according to each subject’s cutaneous sensory threshold.

Since perception of GVS is inherently subjective, we utilized systematic procedures that have been previously utilized in determining subliminal current levels for both GVS and transcranial stimuli [12], [13], [48]. Starting from a basal current level of 20 µA, noisy test stimuli were delivered for 20 s periods with gradual stepwise increases (20 µA) in current intensity until subjects perceived a mild, local tingling in the area of the stimulating electrodes. A threshold value was defined once subjects reported the tingling sensation as performed previously [12], [13], which lasted for the duration of the test stimulus. The current level was then decreased each time by one level until sensation was no longer reported during delivery of test stimulus pulses, and increased by one step in current intensity to confirm threshold. Each delivery of a test stimulus was followed by a period of no stimulation for at least 30 s to preclude a hysteretic effect carrying over to the next test stimulus: after a 20 s of high-frequency deep brain stimulation of the STN, beta rhythms return to baseline 15 s after the stimulus finishes [49]. Subjects were blind to the onset and duration of test stimuli, as well as the threshold-testing scheme.

### EEG Acquisition

We recorded the continuous EEG from 19 scalp electrodes using a Neuroscan Synamps^2^ EEG acquisition system and standard electrode cap (Neuroscan, VA, USA). Electrode impedances were maintained below 10 kΩ using Electro-Gel (Electrode-Cap International, OH, USA). Recording electrodes were positioned according to the International 10–20 EEG System (Figure 1) with one ground electrode and linked earlobe electrodes as reference. Surface electromyographic electrodes were positioned above and below each eye for subsequent artifact removal during analysis [50]. All data were digitized at 1 kHz, and bandpass filtered between 1 and 250 Hz.

### EEG Pre-Processing

EEG data were downsampled to 250 Hz and bandpass filtered between 1 and 50 Hz. We subsequently applied Independent Component Analysis (ICA) to remove common artifacts from the recordings [44], [51]. ICA uses linear combinations of electrodes to derive temporally independent waveforms from a mixed signal. Artifacts due to eye movements, muscle activity and heartbeats are statistically independent from ongoing brain rhythms in the time domain, making ICA ideal for artifact isolation and removal [51]. ICA was performed on concatenated EEG data from pre- and post-stimulus periods, and 15 component activations were extracted. Careful joint inspection of the scalp topography, power spectrum and activity of components allowed for deeming specific components for artifact rejection in the pre- and post-stimulus EEG periods.

We assumed that the source localization of common EEG artifact components (e.g. eye movement, muscle artifact) remained unchanged during the stimulation. We therefore utilized the unmixing matrices from ICA performed on concatenated pre- and post-stimulus periods, and applied those matrices to isolate eye, muscle and cardiac artifacts present in the stimulation periods. The use of the pre- and post-stimulus unmixing matrices also ensured that no bias was introduced into the intrastimulus EEG during ICA artifact removal. Common artifact components were similarly assessed and rejected by thorough joint inspection of the scalp topography, power spectrum and activity of components. EEG data were subsequently reconstructed using all other components.

Since skin has a relatively low resistivity in comparison to the skull, a fraction of the stimulating currents could potentially be directly shunted across the scalp and picked up by the recording electrodes [52]. The issues of EEG data containing stimulus artifacts or of removing too much neural information during artifact rejection pose a central concern with simultaneous electrical stimulation and recording approaches [53], [54]. During the stimulation period, microvolt recordings of biological activity may be overwhelmed by higher-voltage shunted stimuli. In order to remove stimulus-based artifacts from the EEG, we concatenated recordings of the 6 stimulation periods for each subject. To remove the direct effects of shunting, we utilized the linear-based [55], [56] QR decomposition (*qr* function in Matlab). We created an augmented matrix consisting of the EEG (with artifacts removed via ICA) and the temporally-aligned stimulus signal. The QR decomposition of the real matrix *A* computes an orthogonal matrix *Q* and upper triangle matrix *R* such that 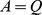. In the current situation, we created the matrix *A* so the first column was the stimulus, and subsequent columns were the concatenated EEG recordings. We then performed the “economy-size” QR decomposition. The rows of *Q* corresponded to the number of time points, and the number of columns corresponded to the number of EEG channels +1 (corresponding to the stimulus). By setting the first row of *R* to zero, to create *R*_0_, then deriving 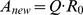, we can obtain the EEG data with the stimulus regressed out. Previously, stimulus-induced artifacts have been removed from potential recordings using a least squares regression [53]. Similarly, we chose the QR decomposition due to: 1) its numerical stability and computational efficiency for a large number of EEG recording channels [45], as well as 2) its proven recognition accuracy of discriminant vectors when applied for feature extraction of high dimensional data [46]. Following rejection of stimulus-induced artifacts, the reconstructed EEG stimulation periods were then divided into non-overlapping, 1-s epochs. Each epoch was then finally inspected to ensure absence of stimulation or other artifacts.

### Power Spectral Analysis

#### Aftereffects of stimulation

In order to investigate whether the effects of GVS potentially have any direct effect on brain rhythms, we analyzed net EEG spectral changes following the highest-level stimulation condition (90% threshold). In one subject, the data was corrupted towards the end of the trial; therefore, we used the first 40 s for all subjects. For the artifact-free pre- and post-stimulus periods from the trial with current level 6, we calculated time-varying changes in power spectral density (PSD) for each electrode channel using a short-time Fourier transform (*spectrogram* function in Matlab, nFFT = 256, window = 125 points, overlap = 62 points). For each window segment, the spectral difference was taken from the post-stimulus minus the pre-stimulus periods, and we applied a one-sided t-test to see whether net spectral changes within a given frequency band were significantly different from the pre-stimulus period. Changes in spectral amplitude were analyzed for each of 5 frequency bands of interest: theta (4–7.5 Hz), low alpha (8–10 Hz), high alpha (10.5–12 Hz), beta (13–30 Hz), gamma (31–50 Hz). Spectrograms were plotted for each electrode channel and show mean spectral changes across all subjects. Since the order of the 6 stimulus levels was pseudorandom and varied for all subjects, the inherent issue of EEG non-stationarity is largely precluded. Significance was determined at *p*<0.05.

#### Effects of stimulation

In order to determine whether GVS effects were associated with ongoing EEG changes, we analyzed the PSD of activity recorded in each electrode during the stimulation period and within the same frequency bands of interest: theta, low alpha, high alpha, beta and gamma. PSD features of activity recorded were calculated for each 1−s epoch of artifact-free data using a fast Fourier transform with 1−s windows (*pwelch* function in Matlab, nFFT = 256, window = 250 points, no overlap). Specifically, we tried to predict current level given the EEG features using multivariate regression:
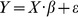
(1)where *Y* was of dimensions 60 (6 current levels × 10 subjects) by 1, *X* was 60 by 95 (19 channels × 5 frequency bands) and 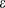
is 95 (19 channels × 5 frequency bands) by 1 and given the EEG feature of the band-limited power over each of the six current levels was removed.

Since, in this case, the number of potential regressors (95) exceeds the number of examples (60), we utilized LASSO regression (*lasso* command in Matlab) [57]. Unlike other methods such as ridge regression or ordinary least squares, LASSO regression puts a sparsity constraint on β so that most values are zero and attempts to find the most informative electrode/band combination of EEG spectral changes to predict current level [57]. The number of regressors selected by the LASSO operator was to give the least predictive error based on a 10-fold cross-validation. Once the regressors were selected, we used robust regression (*robustfit* command in Matlab) to estimate the significance of the individual regressors.

In order to visualize possible non-linear effects of the stimulus, for the significant channels, we plotted actual changes in band-limited power level as a function of stimulus current (in effect, the appropriate column of *X* vs. *Y* in eqn 1).

## Results

Subjects reported a cutaneous sensory threshold at mean RMS current amplitude of 160±110 µA. For the highest-level stimulus condition (current level 6), mean delivered RMS voltage was recorded as 4.6±2.3 V. In consistency with prior observations, subjects additionally did not report perceiving any stimulus during the stimulation periods [13], [36]. Subjects also did not experience postural sway throughout the experiment trials. Some subjects reported feelings of mild dizziness or lightheadness after the experiment.

### Noisy GVS Increased Beta and Gamma Power in the Post-stimulus Period

**Figure 3:**
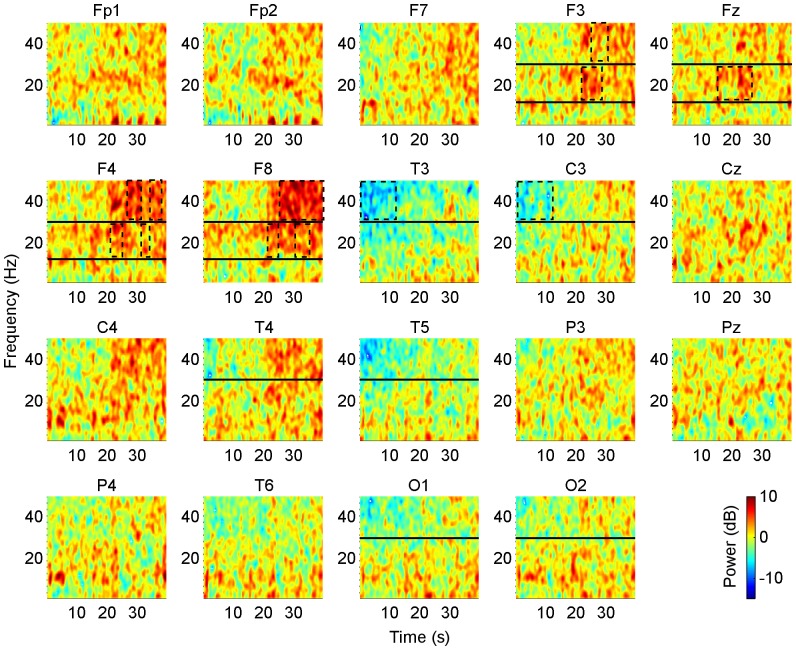
Post-stimulus spectral effects of noisy GVS. Spectrograms of the effects of noisy GVS after stimulation. Spectrograms plot the difference in spectral power in the pre-stimulus subtracted from the post-stimulus periods, thus showing net spectral changes for the first 40 s following the cessation of stimulation. Beta and gamma changes occurred after a marked delay following the end of stimulation. In frontal regions (F3, Fz, F4 and F8), beta power increased significantly starting 18–23 s after stimulation ended, while gamma power in F3, F4 and F8 increased significantly starting 26–27 s after stimulation ended. In lateral electrodes T3 and C3, gamma power was suppressed significantly within the first 10 s immediately following stimulation. For spectrograms of electrodes F3, Fz, F4 and F8, beta and gamma frequency bands are delineated by an upper horizontal black line at 30 Hz and a lower horizontal black line at 12 Hz. For electrodes T3, C3, T4, T5, O1 and O2, the gamma band is delineated by the horizontal black line at 30 Hz. Rectangles outlined in dotted black lines enclose significant spectral changes. Spectral power is reported in dB, as indicated by the colour legend. Significance was determined at *p*<0.05 (see Table S1 for *p* values).

In the post-stimulus period, significant net spectral effects were noted in the spectrograms for electrode channels in frontal and bilateral regions. In electrode channels F3, Fz, F4 and F8, beta power significantly increased starting 18–23 s after the stimulation had ceased (*p = *0.019, 0.021, 0.018, 0.039 respectively, Figure 3). In a similar fashion, gamma power significantly increased starting 26–27 s later in fronto-lateral areas F3, F4 and F8 (*p* = 0.011, 0.037, 0.022 respectively, Figure 3). Overall, we conclude that the significant augmentation in power of beta and gamma rhythms in frontal areas appeared with a brief delay of approximately 20–25 s after stimulation ended, and lasted only several seconds with the strongest effects of gamma suppression in F8 lasting up to 40 s after stimulation stopped. Additionally, we note that the effects were lateralized with power increases occurring predominantly in electrode channels in the right hemisphere. In T3 and C3, transient and mild suppression of gamma power was observed immediately after stimulation stopped during the first 10 s of the post-stimulus period (*p = *0.046, 0.023 respectively, Figure 3). Upon attentive inspection, we observed gamma power suppression occurred immediately after stimulation in lateral and occipital channels T4, T5, O1 and O2, lasting about 5 s (Figure 3); however, this suppression did not reach significance (*p*>0.05). Lastly, since the significant *p* values were not greatly less than the limit (0.05), we conclude that the aftereffects of the stimulation on EEG rhythms, while visually visible across the mean of subjects, were mild and short-lived – presumably due to the weak, subsensory levels of currents delivered (see Table S1 for adjusted *p* values and exact time points in the spectrograms which showed significant spectral changes).

### Stimulation Intensity is Linearly Related with EEG Power Features across Bands

**Figure 4:**
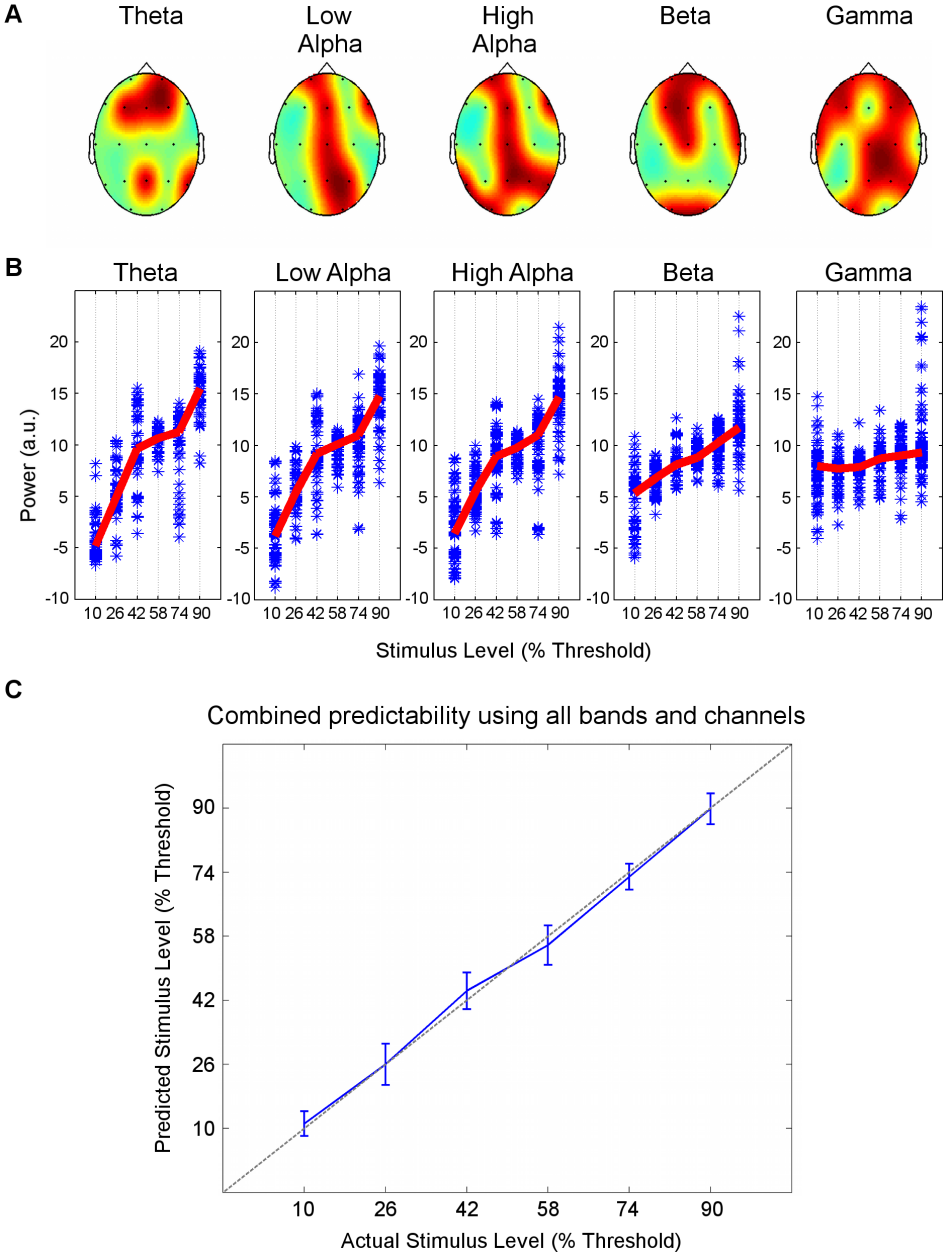
Combined band power in significant channels predicted stimulus intensity in a linear manner. **A.** LASSO regularization identified significant channel/band combinations whose spectral features predicted the stimulus intensity in a linear manner. Significant channels selected from each band LASSO are shown on scalp maps in red. Theta band power was significant in channels Fp2, F3, Fz, F4, Pz, T6, low alpha band power in Fp1, Fz, F8, Cz, Pz, P4, O2, high alpha band power in Fp1, Fz, F8, Cz, Pz, T5 P4, T6, O1, beta band power in Fp1, Fp2, F3, Fz, F8, Cz, T4, O1, O2, and gamma band power was significant in Fp1, Fp2, F7, F3, F4, F8, Cz, Pz, T3, C4, P4, T6, O1. **B.** Spectral power in significant channels were plotted as function of stimulus intensity. The mean spectral power for each subject has been removed. Line plots (red) represent the median spectral values for all significant channels across all subjects. **C.** The ability of EEG features to linearly estimate stimulus intensity when all bands were included was confirmed by plotting predicted estimates against actual values of stimulus intensity. Blue line indicates the stimulus intensity predicted by LASSO-selected EEG estimates whereas the dotted gray line represents an ideal linear relation. Error bars were estimated from leave-one-out cross validation.

In the theta band, the LASSO algorithm identified 6 significant electrode channels in frontal areas (Fp2, F3, Fz, F4), the posterior-midline (Pz) and right lateral side (T6). In the low alpha band, LASSO identified 7 significant electrode channels in frontal areas (Fp1, Fz, F8), the central/midline area (Cz, Pz) and right posterior area (P4, O2). In the high alpha band, LASSO identified 9 significant electrode channels in frontal channels (Fp1, Fz, F8), the central/midline area (Cz, Pz) and bilateral posterior areas (T5 P4, T6, O1). In the beta band, LASSO identified 9 significant electrode channels in frontal areas (Fp1, Fp2, F3, Fz, F8), the central area (Cz), the right lateral side (T4) and occipital areas (O1, O2). In the gamma band, LASSO identified 13 significant channels in frontal areas (Fp1, Fp2, F7, F3, F4, F8), the central/midline area (Cz, Pz) and bilaterally throughout central (T3, C4) and posterior areas (P4, T6, O1). All electrode regions selected by the LASSO operator as related linearly with band power are illustrated in Figure 4A. Significance was determined at *p<*0.05 (see Table S2 for *p* values).

For each frequency band of interest, median spectral power measured in the above significant electrodes for all subjects were plotted as a function of stimulus intensity (Figure 4B). Note that this is the same as plotting the appropriate columns of *X* as a function of *Y* in eqn. 1. When the information from all columns of X (i.e., all bands) was included, plotting *X β* vs *Y* resulted in a linear relation (Figure 4C).

## Discussion

We have shown for the first time, to our knowledge, that noisy GVS influences ongoing EEG activity when applying a simultaneous galvanic vestibular current during resting state with eyes open. Transient changes in spectral features, notably in the beta and gamma bands in frontal regions, were observed after cessation of stimulation, therefore demonstrating that GVS directly modulated brain rhythms. Subsequently, upon analyzing ongoing EEG changes, we observed a dose-dependent relation between stimulation intensity and EEG power spectral features, which measure oscillatory amplitude corresponding to neural synchrony. Such dose dependency has been implicated by previous work, showing that supersensory direct current GVS applied during a visual processing task increased delta power to a greater amplitude than subsensory stimulation [36]. Furthermore, we observed spectral changes in all bands of interest (theta, alpha, beta, gamma) across predominately frontal-parietal electrodes. Therefore, our work suggests that noisy, imperceptible GVS modulates global synchronization of neural oscillatory activity across theta, alpha and – outside of the stimulus frequency – beta and gamma frequency bands with transient aftereffects.

Since we did not measure spectral changes beyond 40 s after stimulation ended, it is unknown whether power changes lasting greater than 40 s were present. However, direct stimulation of the STN for 20 s by deep brain stimulation in Parkinson’s disease has resulted in beta power changes which persist for 15–25 s after stimulation cessation [49]. On this basis, due to the short duration of our weak, transcranial stimulation protocol (72 s), we infer it is unlikely novel post-stimulus spectral changes occurred beyond 40 s. Additionally, whether our observed spectral changes are long-lasting to induce synaptic plasticity is beyond the scope of this paper. In the present study, we largely focused on simultaneous effects of GVS on EEG activity, and discuss the relevance of our results with respect to vestibular processing and neural oscillations.

Consideration must be given to the possibility that our results were influenced by imperfect artifact removal: stimulus currents might have been directly shunted along the scalp to the recording electrodes, and/or the stimulus current may have simply propagated non-specifically throughout brain tissue. However, our conclusions were likely not based on false positive results. First, we demonstrated significant post-stimulus spectral changes immediately after cessation of stimulation, supporting our hypothesis that brain rhythms are directly influenced as a result of stimulation. Secondly, linear regression methods have been proven previously to remove stimulus-related artifacts from potential recordings in rats [53]. The applied QR decomposition method similarly relies on linear transformation with greater computational efficiency and accuracy appropriate for the high number of electrodes [45], [46] to robustly isolate any EEG features that exactly resembled the stimulus. In addition, while the temporal profile of the stimulus may have been altered due to potential capacitive and inductive characteristics of scalp tissue, significant EEG changes were observed in frontal, midline and posterior regions – far from the stimulating electrodes – in both analyses of post-stimulus and ongoing effects. EEG changes were importantly observed in frequency bands greater than the stimulus range of 0.1–10 Hz (i.e., in high alpha, beta and gamma bands). Rather, our results are consistent with the notion that GVS may directly alter firing in vestibular nerve projections and ensuing thalamocortical neural connections [16].

The observed effects on EEG activity may be explained by direct modulation of vestibular processing areas and possibly indirect effects on cortico-cortical connections. GVS is a well-established technique for delivering a weak current that bypasses hair cells and alters firing patterns of vestibular afferent nerves in the same manner as natural stimulation [9], [58]. Since the vestibular nerve runs underneath the mastoids towards brainstem nuclei [16], transmastoidal stimulation has effectively and consistently been shown numerous times to activate vestibular-related subcortical and cortical regions [4], [59]–[62] and elicit appropriate consequences on balance-related functions and ocular movements [63]–[65]. Direct cathodal stimulation of the vestibular end organ depolarizes the transmembrane potential predominantly at the spike trigger zone whereas anodal stimulation inhibits firing [9], [58]. Therefore, depending on the existing neural connections and brain state, externally applied stimulating currents may spread from target regions trans-synaptically to modulate cortical and subcortical activity [66]. This modulation may be based on manipulation of complex thalamocortical loops receiving input from vestibular afferent projections through thalamic relay neurons, such as the pulvinar [3], [8]. Unlike other non-invasive brain stimulation techniques targeting specific cortical areas, GVS therefore has a more direct influence on thalamic processing.

We delivered GVS at imperceptible levels as determined by cutaneous sensory thresholds. While objective measures such as postural sway and eye movements via activation of the vestibulo-ocular reflex would have been potentially useful to establish a definitive threshold, we relied on subjective reporting to determine each subject’s individual sensory threshold to GVS for a number of reasons. Importantly, the noisy waveform of our stimulus is less apt to produce an easily quantifiable measure compared to a DC or sinusoidal stimulus [9]. Postural sway movements tend to the anodal side of stimulation [9] while the eye experiences an ipsiversive ocular torsion with respect to the anodal side of stimulation [67]. Therefore, the noisy nature of the stimulus waveform would inherently preclude accurate measurements of sway or ocular movements. In addition, prior studies have demonstrated that low-level currents (less than 0.5 mA) are insufficient to elicit any other responses [9]. Our results are thus directly related to modulation of ongoing EEG rhythms and are not masked by postural sway, ocular movements, and perception of body rotation, auditory or pain modalities. Third, it was important that subjects were unaware of the stimulation in order to avoid confounding variables due to voluntary attention and/or general arousal via the reticular formation. Careful debriefing after the experiment revealed the subjects did not sense the stimulation at any time throughout the study, which might suggest that the determined threshold had been set inappropriately high. In contrast, if the determined threshold had been set inappropriately low, our ability to detect significant changes in brain rhythms would have been hampered. Here, we used subjective reports as a reliable approach to determine GVS sensory threshold levels as used previously [12], [36], and consequently achieved significant results with the applied subthreshold current intensities. In addition, the question of whether subsensory stimulation was arguably sufficient to modulate EEG rhythms has been addressed recently [36]; the previously demonstrated changes in event-related potentials and spectral power in response to subsensory GVS – identified in the same manner according to cutaneous sensory threshold – refute the possibility of insufficient current levels [36] as do our observed post-stimulus spectral changes.

### Modulation of Synchrony Patterns and Global Oscillatory Networks

Consistent with the view that no single vestibular cortical region exists [3], [4], [8], our results demonstrate that noisy GVS increased the overall amplitude of synchrony patterns in theta, alpha, beta and gamma bands measured throughout frontal, central/parietal, bilateral and occipital electrodes. Prior studies have demonstrated that GVS induced similar broadband spectral changes in delta, theta, alpha and beta bands, throughout frontal, temporal, posterior, occipital electrodes – yet mainly over midline and lateral channels [36]. In comparison to these prior findings, we specifically observed modulation of each band power consistently throughout frontal sites. Differences in results may be attributed to the nature of the stimulus (direct current vs. noisy) and experimental paradigm (resting state vs. visual task-related) [36]. We conclude our results reflect global modulation of synchrony patterns across a broad range of oscillations.

The broadband changes we observed are notably interesting because synchronization of slow and fast frequency oscillations cooperate to mediate various cognitive and behavioural functions. Simultaneous alpha, beta and gamma oscillations integrate and cooperate in attention, working memory and perception [68]. Even theta and gamma oscillations have been shown to be “nested” (i.e., with the amplitude of the faster rhythms phase-locked to the slower oscillation), while temporally coinciding with conscious visual percepts in humans [41]. Of greater interest, integration of theta and gamma synchrony occurred throughout a large-scale prefrontal-parietal network [41]. Similarly, coherence of beta and gamma power throughout a large-scale motor-striate network has been demonstrated to dynamically change throughout a GO-NO-GO motor paradigm [69]. In the present study, we show that noisy GVS significantly increased the amplitude of theta, alpha, beta and gamma power in prefrontal and posterior (parietal and/or occipital) regions. The fact that noisy GVS modulated alpha, beta and gamma power in occipital electrodes O1 and O2, which corresponds to the striate cortex [70], is not surprising. GVS has been previously demonstrated to enhance visual processing, such as visual memory recall in normal subjects [12] and spatial processing performance in stroke patients [15], [17], [71], [72]. A more remarkable observation, however, is that theta, alpha and gamma power were significantly modulated throughout prefrontal and parietal (Pz/P4) electrodes, which correspond to the precuneus [70]. Strong connectivity between prefrontal cortex and precuneus is well-established [73], [74], with the latter region being particularly important in gating thalamocortical activity [74] and various cognitive domains, such as episodic memory retrieval, visuo-spatial imagery and self-awareness [73]. With the functional role of large-scale synchrony patterns in mind, our results showing EEG modulation by noisy GVS may explain the previously reported phenomenological effects on cognition and behaviour.

Modulation of large-scale networks by noisy GVS may in fact reflect an influence on global information flow between cortical neurons oscillating at similar frequencies. Functional networks in the brain may demonstrate small-world properties (i.e., highly clustered nodes of locally-connected interneurons that are inter-regionally connected) [75]. In order to achieve specific behavioural goals for perception, cognition and action, communication among nodes are dynamically controlled or “gated” for optimal network configuration. Synchronization of oscillatory signals is hypothesized to serve as the dynamic gating mechanism between functional nodes [40], [42], [76]. The periodicity of synchrony patterns determines neuronal responsiveness. Maximal responsiveness occurs around the depolarizing or excitability peaks, thereby facilitating effective communication between neuronal groups when the timing of excitability peaks is coordinated. Conversely, information flow is minimal when oscillations are not synchronized, or excitability peaks misalign with troughs [77]. Dynamic modulation of neural synchronization patterns is therefore suggested to be important for information processing in functional networks [40], [69], [78], [79]. In light of this, individual activity of brain regions may not be so characteristic of networks so much as the dynamic nature of their “links”, which are mediated by synchrony over multiple frequency bands [69]. Since we show that noisy GVS is linearly related with broadband synchrony changes throughout large-scale networks, our results may therefore pose noisy GVS as a relevant tool for modulating and understanding brain networks.

### Effects of Noisy Stimulation

Stochastic facilitation (a broader term for “stochastic resonance”) may be a putative mechanism through which noisy GVS modulates the amplitude of EEG synchrony. In this model, biologically relevant noise may enhance neural information processing and computational goals [37]. For example, stochastic facilitation has been suggested as the mechanism through which noisy GVS improves visual memory while constant current GVS does not [12]. If a non-linear dynamical system (e.g., a neuron) is partially depolarized by a subsensory stimulus, adding random noise to a weak stimulus may render the signal detectable via random intermittent depolarization [37], [80]. Therefore, broadband sensory noise, even at high frequencies, may enhance synchronization at both intra- and inter-regional cortical levels [40]. A similar framework may apply to our results: noisy vestibular stimulation may engage synchronization of neuronal assemblies [80]. The particular 1/*f* power density of the applied stimulus may specifically recruit more global, integrative networks at slower oscillations, which perturb local, higher-frequency oscillations in rhythm-generating networks of GABA interneurons [81]. This is contrast to sinusoidal transcranial stimulation which has been shown to modulate LFPs in widespread cortical areas albeit entraining neural oscillations instead, driving them at a particular frequency [53]. Stochastic facilitation is consequently a proposed mechanism to explain the observed effects across all EEG bands of interest, as opposed to solely within the frequency range of the stimulus (<10 Hz).

In support of this view, others have proposed stochastic facilitation as an explanation for their observations of the effects of noisy GVS. For example, noisy GVS enhanced GABA release and altered neurotransmission within the substantia nigra in both unlesioned and 6-hydroxydopamine hemilesioned Parkinsonian rats [22]. Notably, while white noise stimulation has also been shown to sensitize other systems, such as the baroreflex response, 1/*f* noisy stimulation is more optimal and effective in doing so [47]. The authors of the study suggested that 1/*f* noise “kicks” the system out of insensitive fixed states [47]; therefore, putting the brain in a more metastable (i.e., dynamic) state [38]. Accordingly, the post-stimulus changes we observed after the highest-level of current stimulation may reflect a greater dynamical state. In analyzing the weak, transient aftereffects of noisy GVS, much to our surprise, most significant were the delayed increases in beta and gamma synchronization in frontal electrodes following 20–25 s after stimulation ceased. Similar to how beta synchronization transiently rebounds after a movement or after a behavioural decision to reflect a new network state [35], [82], the delayed beta and gamma synchronization may reflect greater network dynamics. One potential caveat concerning stochastic facilitation, however, is that the output performance depends upon the noise magnitude. This dependency occurs in a relation that follows an inverted U shape, indicating it is possible to overshoot optimal levels of performance [83]. Therefore, while stochastic facilitation is a strong candidate to explain our observed effects, more work is needed to elucidate whether varying noise levels may differentially affect our results.

Lastly, despite that stochastic facilitation suggests that the neuron is a non-linear dynamical system [83], this does not invalidate the possibility of detecting linear effects between EEG spectral features and GVS current intensity. Stochastic facilitation acts at individual neurons whose firing responses are influenced in a non-linear manner. EEG oscillations, on the hand, represent a sum of added and cancelled vector signals, which may be influenced by externally applied GVS in a linear fashion.

### Conclusions

In summary, we demonstrate clear broadband spectral changes during and after stimulation with noisy GVS. The changes we observed were widespread throughout a global assembly of frontal, central/parietal, occipital and bilateral regions. Consistent with our present observations, prior scalp EEG studies have observed broadband spectral changes in normal, healthy subjects, although during visuomotor task performance [84]. Nonetheless, we expected to see changes mainly in beta rhythms, especially based on previous accounts of noisy, imperceptible GVS ameliorating motor function in Parkinson’s disease [20], [21]. In Parkinson’s disease, exaggerated beta synchrony propagates throughout basal ganglia-thalamocortical circuitry, accompanies motor symptoms [43], [85], [86], and is adjusted to a functional range of dynamics by therapies, such as deep brain stimulation of the STN and dopaminergic medication [31]. Since beta synchronization supposedly characterizes a normal state of cortical-basal ganglia networks during sensorimotor processing [35], we therefore speculate that noisy GVS will adjust maladaptive modulatory oscillatory dynamics of the same networks that may be stuck in a particular state. Our results may be especially relevant towards the recently reported motor improvement in a Parkinsonian rodent model [22] and patients [20], [21]; yet, further work will need to confirm this.

## Supporting Information

Table S1 ***p*** **values of post-stimulus spectral changes.** * Only significant *p* values (<0.05) are reported, indicating whether the power of a given band in the post-stimulus EEG was different from the pre-stimulus EEG. † A one-sided t-test was performed on the power difference between post- and pre-stimulus EEG data at each Fourier transform window. Reported *p* values are an average of those found significant within the identified time span by one-sided t-tests. Only significant values spanning a time period of at least 2 s were considered.
(DOCX)

Table S2 **Electrode channels and recorded band power determined by LASSO to predict a linear relation between EEG features and stimulus intensity.** Only significant *p* values (*p*<0.05) are reported. All other *p* values were not significant and are denoted by −.
(DOCX)
