# Supplementary material for: Noisy Galvanic Vestibular Stimulation Modulates the Amplitude of EEG Synchrony Patterns
Source: PLoS One. 2013 Jul 18;8(7):e69055. doi: 10.1371/journal.pone.0069055 (PMC3715484; doi:10.1371/journal.pone.0069055)
Supplement: Table S1 — p values of post-stimulus spectral changes. * Only significant p values (<0.05) are reported, indicating whether the power of a given band in the post-stimulus EEG was different from the pre-stimulus EEG. † A one-sided t-test was performed on the power difference between post- and pre-stimulus EEG data at each Fourier transform window. Reported p values are an average of those found significant within the identified time span by one-sided t-tests. Only significant values spanning a time period of at least 2 s were considered. (DOCX) [file pone.0069055.s001.docx]

| Electrode Channel | Frequency Band | Time (s) | *p* value *† |
| --- | --- | --- | --- |
| F3 | Beta | 23-27 | 0.019 |
| Fz | Beta | 18-26 | 0.021 |
| F4 | Beta | 22-23, 34-36 | 0.018, 0.026 |
| F8 | Beta | 22-25, 31-34 | 0.039, 0.017 |
| F3 | Gamma | 26-28 | 0.011 |
| F4 | Gamma | 27-31, 36-37 | 0.037, 0.030 |
| F8 | Gamma | 26-40 | 0.022 |
| T3 | Gamma | <10 | 0.046 |
| C3 | Gamma | <10 | 0.023 |
