# Supplementary material for: Noisy Galvanic Vestibular Stimulation Modulates the Amplitude of EEG Synchrony Patterns
Source: PLoS One. 2013 Jul 18;8(7):e69055. doi: 10.1371/journal.pone.0069055 (PMC3715484; doi:10.1371/journal.pone.0069055)
Supplement: Table S2 — Electrode channels and recorded band power determined by LASSO to predict a linear relation between EEG features and stimulus intensity. Only significant p values (p<0.05) are reported. All other p values were not significant and are denoted by −. (DOCX) [file pone.0069055.s002.docx]

| Electrode Channel | Theta  (4-7.5 Hz) | Low Alpha  (8-10 Hz) | High Alpha  (10.5-12 Hz) | Beta  (13-30 Hz) | Gamma  (31-50 Hz) |
| --- | --- | --- | --- | --- | --- |
| Fp1 | − | 4.49E-09 | 3.16E-06 | 3.80E-14 | 9.68E-09 |
| Fp2 | 7.84E-07 | − | − | 1.37E-05 | 3.31E-12 |
| F7 | − | − | − | − | 4.56E-16 |
| F3 | 5.06E-16 | − | − | 2.99E-17 | 2.05E-17 |
| Fz | 2.19E-15 | 4.82E-14 | 7.46E-13 | 8.66E-17 | − |
| F4 | 5.64E-14 | − | − | − | 2.94E-13 |
| F8 | − | 1.77E-05 | 2.74E-14 | 4.78E-12 | 3.50E-11 |
| T3 | − | − | − | − | 3.70E-09 |
| C3 | − | − | − | − | − |
| Cz | − | 2.97E-11 | 1.44E-8 | 4.46E-13 | 3.54E-13 |
| C4 | − | − | − | − | 4.64E-11 |
| T4 | − | − | − | 2.40E-07 | − |
| T5 | − | − | 6.22E-07 | − | − |
| P3 | − | − | − | − | − |
| Pz | 5.46E-14 | 5.71E-10 | 2.75E-09 | − | 4.24E-16 |
| P4 | − | 1.61E-04 | 6.13E-11 | − | 1.01E-10 |
| T6 | 4.03E-09 | − | 1.56E-03 | − | 1.02E-13 |
| O1 | − | − | 2.20E-12 | 5.80E-09 | 3.48E-11 |
| O2 | − | 9.43E-07 | − | 1.77E-08 | − |
